# Addressing poverty through disease control programmes: examples from Tuberculosis control in India

**DOI:** 10.1186/1475-9276-11-17

**Published:** 2012-03-26

**Authors:** Vishnu Vardhan Kamineni, Nevin Wilson, Anand Das, Srinath Satyanarayana, Sarabjit Chadha, Kuldeep Singh Sachdeva, Lakbir Singh Chauhan

**Affiliations:** 1 International Union Against Tuberculosis and Lung Diseases (The Union), South-East Asia Regional Office, New Delhi, India; 2 Central TB Division, Directorate General of Health Services, Ministry of Health and Family Welfare, Government of India, New Delhi, India; 3 Technical Advisor, International Union Against Tuberculosis and Lung Disease (The Union), The Union South-East Asia Office, C-6, Qutub Institutional Area, New Delhi 110016, India

**Keywords:** Tuberculosis control, Poverty, India

## Abstract

**Introduction:**

Tuberculosis remains a major public health problem in India with the country accounting for one-fifth or 21% of all tuberculosis cases reported globally. The purpose of the study was to obtain an understanding on pro-poor initiatives within the framework of tuberculosis control programme in India and to identify mechanisms to improve the uptake and access to TB services among the poor.

**Methodology:**

A national level workshop was held with participation from all relevant stakeholder groups. This study conducted during the stakeholder workshop adopted participatory research methods. The data was elicited through consultative and collegiate processes. The research study also factored information from primary and secondary sources that included literature review examining poverty headcount ratios and below poverty line population in the country; and quasi-profiling assessments to identify poor, backward and tribal districts as defined by the TB programme in India.

**Results:**

Results revealed that current pro-poor initiatives in TB control included collaboration with private providers and engaging community to improve access among the poor to TB diagnostic and treatment services. The participants identified gaps in existing pro-poor strategies that related to implementation of advocacy, communication and social mobilisation; decentralisation of DOT; and incentives for the poor through the available schemes for public-private partnerships and provided key recommendations for action. Synergies between TB control programme and centrally sponsored social welfare schemes and state specific social welfare programmes aimed at benefitting the poor were unclear.

**Conclusion:**

Further in-depth analysis and systems/policy/operations research exploring pro-poor initiatives, in particular examining service delivery synergies between existing poverty alleviation schemes and TB control programme is essential. The understanding, reflection and knowledge of the key stakeholders during this participatory workshop provides recommendations for action, further planning and research on pro-poor TB centric interventions in the country.

## Introduction

India is the highest tuberculosis (TB) burden country in the world, accounting for nearly one-fifth or 21% of all tuberculosis cases [1]. In 2009, out of the estimated global annual incidence of 9.4 million TB cases, nearly 2 million cases were estimated to have occurred in India [1,2]. India's Revised National TB Control Programme (RNTCP), based on the internationally recommended Directly Observed Treatment Short-course (DOTS) strategy launched in 1997 implemented a phased expansion achieving nation-wide programme coverage in March 2006. RNTCP covers over a billion population (1,164 million) across 632 districts in 35 states and Union territories, and has initiated more than 12.8 million TB patients on treatment, saving an additional 2.3 million lives [2]. RNTCP has sustained new sputum positive (NSP) case detection rates of over 70% and treatment success rates over 85% nationally since 2007, in line with global targets for TB control [2]. TB mortality and prevalence in the country has witnessed a reduction compared with 1990 figures, indicating progress towards achieving TB related targets of the United Nations Millennium Development Goals (MDG) [2].

The World Bank defines international poverty as people living below US$ 1.25 per day [3]. Of the 22 TB high-burden countries accounting for 81% of the global TB burden, 10 countries are categorised under the 'low income category' (GNI < US $ 995), and 9 countries under lower middle income category (GNI US $ 996-3,945), as per World Bank benchmarks for income group classification [1,4]. Within these already poor countries, there are significant disparities compounded by large populations that are densely distributed and with restricted access to basic health services. India is currently ranked 119 out of 169 countries on human development, with 41.8% of population living below the international poverty line [5]. Planning Commission of India (PCI) reports estimate the poverty headcount ratio, assessed based on the national sample surveys to be at 37% in India (rural and urban) [6]. Multidimensional poverty index (MPI) analysis using the developmental parameters of health, education and standard of living reveal that 8 Indian states (West Bengal, Rajasthan, Chhattisgarh, Jharkhand, Odisha, Uttar Pradesh, Madhya Pradesh and Bihar) with a combined population of 421 million live in poverty comparable to the 26 poorest African countries that have a cumulative population of 410 million [7]. That deprivation of health is the second most significant contributor to overall poverty in India after living standard provides ample indication of the prevailing poverty-health co-relation [7]. It is often the poor and socially excluded groups that are disproportionately exposed to ill-health and are unlikely to receive care.

The linkages between poverty and TB are established historically with tuberculosis more commonly occurring in poor people [8]. Socio-economic determinants such as poverty, overcrowding, food insecurity and malnutrition while identified as proximate risk factors facilitating transmission of infection and disease, are also responsible for inequities in accessing TB care [9]. Poor were two times more likely to have TB, three times less likely to access TB care, four times less likely to complete treatment and many times more likely to incur impoverishing payments for TB care [10]. The impact of TB on poor included loss of income, stigmatisation and homelessness, more acute in women and children [10]. Furthermore, TB patients reduced ability to work is projected to incur a 20-30% loss in their annual wages [11]. These socio-economic determinants amplify health inequities within populations particularly among the poor and vulnerable groups.

In India, free diagnostic and treatment services provided under RNTCP are designed to benefit the poor and vulnerable groups of the society. However, recent evidence from community based Knowledge, Attitudes and Practices (KAP) survey suggests that people 'most in need' of free services were not accessing or utilising these services, and that a significant proportion of TB patients, illiterate and from low income rural households were being diagnosed and treated outside the DOTS/RNTCP system, and incurring expenditure [12]. RNTCP is moving in to the next phase of programming that includes promoting the concept of 'Universal Access to TB Care'. The implementation of this concept is expected to allow all TB patients in the community to have early access to good quality TB diagnosis and treatment services. One of the key challenge in achieving this objective is addressing reasons behind inter/intra-district disparities in programme performances, with problems persisting more so in the poorer and backward districts of the country. Recognising the pivotal role of TB control in alleviating poverty, the World Health Organisation-Stop TB Partnership established a sub working group on TB and Poverty, the secretariat for this sub group more recently located in a TB high-burden country - India. The role of the secretariat is to coordinate the work of the subgroup and to promote poverty centric TB activities, engaging policy makers, practitioners, non-governmental organisations and other stakeholders in setting the foundations for development of specific tools, guidelines and activities that are aimed at increasing the access of the poor to TB services.

There is very limited information in published literature on what specific measures/efforts are undertaken by the TB Control Programmes to make the services accessible to TB patients among the poor. Secondly there is also limited information on whether the services that are made available are utilised by the poor. Through this paper, we attempt to present these two issues with respect to TB control Programme in India. In addition we also highlight key issues relating to TB control and poverty that emerged during the consultative workshop on 'Poverty and TB'.

## Methods

It is well acknowledged that participatory research (PR) in its four modes: contractual, consultative, collaborative and collegiate if systematically applied in to practice will provide reliable and valid research results that will enhance knowledge for action [13]. This study adopted the participatory research method with focused stakeholder involvement comprising of consultative and collegiate processes.

The consultative method aimed at eliciting 'knowledge for action' information with appropriate recommendations emanating from participant's practical field experiences; while the collegiate methodical approach allowed both the facilitators and participants with varying skills to be involved in a mutual learning experience. The focus of the methods used was to elicit the information on understanding equity issues within the framework of tuberculosis control programme in India and to outline the mechanisms to improve the uptake and access to TB services among the poor. This workshop funded by The Centers for Disease Control aimed at examining pro-poor initiatives adopted in tuberculosis control programme in India and also discuss equity-mapping by adopting participatory techniques involving stakeholders during the 2 day iterative workshop. Workshop on TB and poverty was organised in Gurgaon, India on 29-30 October, 2010, by the Secretariat of the WHO Stop TB Poverty sub working group hosted by The Union's South-East Asia regional office located in New Delhi, India.

The participants (n = 31) were from a wide range of stakeholders that included RNTCP National Programme Manager and TB programme managers representing 5 Indian states with a higher proportion of people living below poverty line: Bihar, Jharkhand, Madhya Pradesh, Chhattisgarh, and Uttarakhand. Two relatively not-so-poor states consisting of poverty afflicted urban slums, Tamil Nadu, Haryana and Union territory-Chandigarh also participated in the workshop. The selection of states was based on existing poverty levels and TB burden as identified by the RNTCP programme in India. In addition, representatives from multilateral and non-governmental agencies, civil society and media: World Bank-India, World Vision-India, International Union Against TB and Lung Disease (The Union), WHO-RNTCP consultants network, Partnership for TB Care and Control in India (national civil society partnership), and Citizen News Service participated in the study.

The first day of the participatory workshop allowed for overview discussions on poverty and TB at country level, followed by structured presentations and discussions on pro-poor TB initiatives in the represented states/union territories (n = 6) Jharkhand, Bihar, Chhattisgarh, Tamil Nadu, Chandigarh and Uttarakhand. A pro-poor initiative was defined as any measure that is introduced/implemented and expected to specifically benefit the TB patients who are poor. The end-session on Day 1 allowed for specific inputs from participants in to the PR design with inputs identifying working groups and segregating the participants in to 3 thematic groups. The second day of the participatory group work allowed participants to deliberate on the thematic areas relating to pro-poor approaches; existing strategies and pro-poor recommendations; and lastly equity mapping to measure equity in TB control. Facilitated discussions in the group work were aimed at identifying recommendations that were likely to be later examined in a systematic manner at the level of RNTCP programme and other TB projects having scope for implementing pro-poor interventions in their target populations. Results from each group were discussed among all the participants in obtaining inputs and final consensus contributing to an improved knowledge and understanding on pro-poor initiatives within the framework of TB control.

In addition, the research study also factored information from the following primary and secondary sources: literature review examining poverty headcount ratios and BPL population in Indian states (Table 1) and qualitative and quantitative profiling assessments to identify poor, backward and tribal districts as identified by the TB programme in India. No ethical approval was required for this study.

**Table 1:** States with Poverty Headcount ratio and BPL Population in India

Sl no	State	Poverty Headcount ratio (%)*	BPL Population (million)**
1	Orissa	57.2	17.84
2	Bihar	54.4	36.91
3	Chhattisgarh	49.4	9.09
4	Madhya Pradesh	48.6	24.96
5	Jharkhand	45.3	11.63
6	Uttar Pradesh	40.9	59.00
7	Tripura	40.6	0.63
8	Maharashtra	38.1	31.73
9	Manipur	38.0	0.39
10	Assam	34.4	5.57
11	Rajasthan	34.4	13.48
12	West Bengal	34.3	20.83
13	Karnataka	33.4	13.88
14	Uttaranchal	32.7	3.59
15	Gujarat	31.8	9.06
16	Arunachal Pradesh	31.1	0.20
17	Sikkim	31.1	0.11
18	Andhra Pradesh	29.9	12.61
19	Tamil Nadu	28.9	14.56
20	Goa	25.0	0.20
21	Haryana	24.1	3.21
22	Himachal Pradesh	22.9	0.63
23	Punjab	20.9	2.16
24	Kerala	19.7	4.96
25	Meghalaya	16.1	0.45
26	Mizoram	15.3	0.11
27	Pondicherry	14.1	0.23
28	Jammu & Kashmir	13.2	0.58
29	Delhi	13.1	2.29
30	Nagaland	9.0	0.39
	**All India**	**37.2**	**301.28**

Source: *Planning commission of India, Tendulkar committee report, 2009

**BPL Population of each state was obtained from the Planning Commission press note on poverty estimates for 2004-05. The note is available at http://planningcommission.gov.in/news/prmar07.pdf

## Results and discussion

Expanding tuberculosis control in India has led to a total health benefit of 29.2 million disability adjusted life years (DALYs) and generating a return of US$ 115 for every 1 US$ spent [14]. Referring to the study by Goodchild et al. (2011), the RNTCP programme manager highlighted that an effective TB control programme was already a positive step towards reducing economic 'health shocks' and poverty alleviation. The pro-poor measures identified by the TB control programme managers was additionally facilitated through linking identified poor TB patients to poverty-alleviation schemes provided by the central and state government. RNTCP had introduced targeted pro-poor approaches by designing specific tribal action plans in tribal areas/districts to address the special needs of tribal people, and to compensate for their loss of wages during treatment. Incentives were paid to tribal patients in hard-to-reach areas to support transportation to TB facilities and on successful completion of treatment. Innovative interventions associating poor patients with anti-poverty schemes to obtain financial and nutritional support had been piloted across 5 districts in West Bengal by CARE in close collaboration with State TB Cell. Table 2 outlines the 144 poor, backward and tribal districts as documented by the RNTCP programme where pro-poor approaches were currently implemented. State programme managers representing Jharkhand, Bihar, Chhattisgarh, Tamil Nadu, Chandigarh and Uttarakhand provided broad insights into their work addressing poverty within the framework of RNTCP.

**Table 2:** Profile of Poor, Backward and Tribal districts, RNTCP programme

S No	State	Districts (n)	Districts
1.	**Arunachal Pradesh**	**3**	Changlang **,Lohit **,Tirap †

2.	**Bihar**	**32**	Araria **,Aurangabad-BI **,Banka, **,Begusarai **, Bhagalpur **,Bhojpur **,Darbhanga **,Gaya **, Gopalganj **,Jamui **,Jehanabad **,Kaimur **, Katihar **,Khagaria **,Kishanganj **,Lakhisarai **, Madhepura**,Madhubani **,Munger**, Muzaffarpur **, Nalanda**,Nawada**,Pashchim Champaran **, Purba Champaran **,Purnia **, Saharsa **,Samastipur **, Saran **,Sheikhpura **,Sitamarhi **,Supaul **,Vaishali **

3.	**Chhattisgarh**	**4**	Kawardha **, Koriya **, Raigarh-CG **, Surguja †

4.	**D & N Haveli**	**1**	Dadra & Nagar Haveli †

5.	**Haryana**	**2**	Kaithal **, Mewat **

6.	**Himachal Pradesh**	**1**	Hamirpur-HP **

7.	**Jharkhand**	**19**	Chatra **, Deoghar **, Dumka **,Giridih **,Godda **, Gumla†, Hazaribagh**,Jamtara**,Khunti†,Kodarma**,Lathehar **, Pakaur **,Palamu **,Purbi Singhbhum †,Ramgarh**, Ranchi †,Sahibganj **,Saraikela-Kharsawan **,Simdega **

8.	**Karnataka**	**3**	Bidar **,Gulbarga **, Yadgiri **

9.	**Madhya Pradesh**	**24**	Alirajpur†,Balaghat**,Barwani†,Betul**,Burhanpur**, Chhatarpur**,Chhindwara**,Damoh **, Dhar †, Dindori †, Harda**,Hoshangabad**,Jhabua†,Khandwa**, Khargone**,Mandla †, Narsinghpur **,Panna **,Raisen **, Sagar **,Sehore **, Seoni **,Tikamgarh **,Vidisha **

10.	**Maharashtra**	**11**	Aurangabad-MH **,Bid **,Buldana **,Gadchiroli ** Hingoli **,Jalna **,Latur **,Nanded **,Osmanabad ** Parbhani **,Yavatmal **

11.	**Orissa**	**10**	Balangir **,Gajapati †,Kalahandi **,Kandhamal †,Koraput †, Mayurbhanj †,Nabarangapur†,Nuapada†, Rayagada†, Sundargarh †

12.	**Rajasthan**	**2**	Banswara †,Dungarpur †

13.	**Sikkim**	**2**	South Sikkim **,West Sikkim **

14.	**Uttar Pradesh**	**26**	Bahraich **,Banda **,Barabanki **,Basti **,Bijnor ** Budaun **,Fatehpur **,Hamirpur-UP **,Hardoi ** Jalaun**,Jhansi**,Jyotiba Phule Nagar**, Kanpur Dehat **,Lalitpur **,Maharajganj **,Mahoba **, Mau **,Moradabad **,Pilibhit **,Pratapgarh **, Rae Bareli **,Sant Kabir Nagar **,Shravasti **, Siddharthnagar **,Sitapur **,Unnao **

15.	**West Bengal**	**4**	Darjiling **,Jalpaiguri **,Koch Bihar **,Maldah **

	**Total**	**144**	

**- Poor and backward districts, † - Poor, backward and tribal district;

Data source--RNTCP Performance report, Q1 2011

The approaches identified as pro-poor in Jharkhand state included collaboration with private providers to improve access to diagnostic services through establishment of sputum collection centres, facilitating community based care, and involving Accredited Social Health Activists (ASHA),^a ^NGOs, Private Practitioners, informal health providers^b ^and community volunteers as DOT Providers to improve access to treatment services. Interventions also include engaging community and faith based organisations for ACSM implementation, sensitising community groups such as Village Health & Sanitation Committee (VHSC),^c ^organising PRI meetings and Patient- Provider meeting so as to contribute towards improving care for the poor in Jharkhand state. Table 3 summarises the key pro-poor approaches currently implemented across states represented in the workshop.

**Table 3:** Summary of pro-poor approaches for TB care, RNTCP

State	Pro-poor approaches
**Jharkhand**	• Collaboration with private providers
	• Improving access to diagnostic services
	• Facilitating community based care
	• ASHA, NGOs, Private Practitioners, Rural Medical Practitioners and community volunteers as DOT Providers to improve access to treatment services
	• Involving community and faith based organisations for ACSM implementation
	• Involving VHSC, PRI members, facilitating Patient-Provider meetings to improving care for the poor

**Bihar**	• Mapping and identifying vulnerable groups, flood affected areas and displaced (refugee) populations
	• Regular health gatherings to promote TB awareness
	• Non-governmental organisations providing nutritional/food assistance to patients
	• Advocating for a legal framework to protect against loss of employment
	• District TB managers coordinating with the local social welfare department
	• Public-private mix to improve access of the poor to TB services

**Chhattisgarh**	• Involving unqualified practitioners in rural areas and urban slums in TB control
	• Involving NGOs and public sector units (SAIL, NTPC, Railways and COAL)
	• Targeting special population groups - establish TB facilities targeting refugee communities and prisoners
	• Implementation of a state specific tribal action plan
	• Involvement of Mitanins (ASHAs) in suspect referrals and as DOT providers

**Uttarakhand**	• Involving CBOs for RNTCP sputum collection and transportation schemes
	• Seriously ill TB patients provided grant assistance from the State Illness relief fund
	• Rastriya Swasthiya Bhima Yogana (RSBY) reimbursement for those TB patients who required hospitalisation
	• Sudurvarti Sahayaks from CM's Sudurvarti Gram Yojana involved TB services
	• No user charges for the poor utilising X-ray facilities for TB diagnosis

**Tamil Nadu**	• Engaging private providers in TB control activities
	• Increasing patient engagement in DOTS/Community based care
	• Improving drug supply management to improve drug accessibility to the vulnerable groups
	• Collaborating with civil society organisations in ACSM activities to promote the awareness of TB control in the vulnerable population
	• Collaboration with private/NGO partners in areas requiring additional diagnostic services

**Chandigarh**	• Engaging private sector and NGOs,
	• Targeting missing cases in poverty pockets in urban areas
	• Availability/accessibility of diagnostic services, and improving availability of drugs to the poor. Reaching the unreached and refugee communities, prisoners and tribal populations (32% in state) by ACSM and involvement of ASHAs

TB Programme in Bihar had initiated geographical mapping and identifying vulnerable groups (marginalised/discriminated groups) based on their residential location. Mapping exercise was inclusive in identifying flood affected areas, urban slums and displaced populations. Under the aegis of National Rural Health mission (NRHM),^d ^regular health gatherings were organised in close collaboration with the District TB centres. Non-governmental organisations such as Damien Foundation and LEPRA India were providing nutritional/food assistance to patients who were sole household earning members. Pro-poor initiatives targeting social barriers included advocating for a legal framework to protect against loss of employment, and investigating attitude and behaviour of health staff towards patients. The state was exploring mechanisms to address geographical barriers that included advocating with the national programme to relax eligibility norms for the creation of diagnostic services and increase their number within a population. District TB programme managers were participating in meetings of the local social welfare department. Tracking out-migration, mapping of vulnerable groups and increasing numbers of diagnostic services within the population were some of the pro-poor initiatives.

The approaches identified as pro-poor in the state of Chhattisgarh included involving informal health providers in rural areas and urban slums to promote TB suspect referral and in DOT provision for diagnosed TB patients, involve NGOs and public sector units (SAIL, NTPC, Railways and COAL) in TB control to enhance access of TB services to the poor. Vulnerable population groups have received special attention with TB facilities established in Mainpat Tibetan camps and Mana Bangladeshi camps targeting refugee communities from Tibet and Bangladesh, and TB facilities have also been set up in Bilaspur and Raipur central jails to provide services closer to prisoners. Implementation of a state specific tribal action plan providing transportation incentives for patients from tribal communities to access diagnosis and treatment services, and involving ASHAs in suspect referrals and as DOT providers were among other poverty centric initiatives aimed at reaching the unreached.

Uttarakhand state has identified new community-based organisations (CBOs) for sputum collection and transportation schemes under RNTCP. TB patients who were seriously ill were at times provided grant assistance from the State Illness relief fund. Rastriya Swasthiya Bhima Yogana (RSBY)^e ^reimbursement was made available for poor TB patients who required hospitalization. Sudurvarti Sahayaks from CM's Sudurvarti Gram Yojana were also involved in providing TB services. User charges were not levied from the poor when they utilised X-ray facilities for TB diagnosis. X-ray diagnosis is an important step in the TB diagnostic algorithm and costs usually associated with this test is also a barrier to its use.

Pro-poor approaches in Tamil Nadu state included engaging private providers in TB control activities, increasing patient engagement in DOTS/Community based care and improving drug supply management to improve drug accessibility to the vulnerable groups. State TB programme was presently collaborating with civil society organisations in implementing ACSM activities to promote the TB awareness in vulnerable populations. In areas where need based analysis identified requirement of additional diagnostic services, collaboration with private/NGO partners was explored to provide services in alignment with RNTCP public-private mix schemes. In order to improve community engagement in DOTS and encouraging community based care, DOT providers were paid an honorarium at the end of the treatment that served as an incentive as well as a motivating factor to continue as DOT providers.

Poverty-centric activities in Chandigarh included engaging private sector and NGOs, targeting missing cases in poverty pockets, examining availability/accessibility of diagnostic services and improving availability of drugs to the poor. The programme was striving to reach the unreached and refugee communities, prisoners and tribal populations (32% in Union territory) through TB Advocacy, Communication and Social Mobilisation and involvement of ASHAs. Participants from represented Indian states also acknowledged the availability of social welfare schemes like public distribution system for distributing essential food commodities to the economically weaker sections, nutrition within the ambit of rural development departments, and various poverty alleviating social welfare schemes relating to self-employment, NREGA^f ^in the country. The RNTCP national programme manager added that the Global Fund to fight AIDS, TB and Malaria (GFATM) Round 9TB project provides a unique opportunity for state/districts to work closely with the civil society organisations (CSO) to link the poor to social welfare schemes. Ensuring optimal implementation of ACSM strategy, strengthening DOT decentralisation, addressing geographic and economic barriers, and developing innovative incentives to address the access bottlenecks were few of the key recommendations for action emanating from the deliberations. Table 4 highlights participants' deliberations on knowledge gaps and the various options available to address poverty through tuberculosis control. The stakeholders identified pro-poor indicators such as proportion of patients linked to the social welfare schemes, proportion of patients living below poverty line, proportion of patients belonging to primitive tribal groups as few of the key indicators that were needed to be examined in sub-district level equity mapping exercises. Table 5 illustrates workshop deliberations on equity related indicators that may be examined and used to measure progress towards addressing poverty through TB control. Though the poverty reduction activities currently in practice were listed, there was limited information on whether the listed activities are reaching the poor, how much money is spent towards implementing the activities and sustainability of such activities. The immediate and later impact of these pro-poor strategies was also not known to the participants.

**Table 4:** Existing strategies and recommendations made during the Working group deliberations

Current strategies	Proposed recommendations
ACSM strategy for TB control available; Funds available for District level programme managers to implement ACSM activities	Ensure optimal implementation of ACSM strategy; messages to reach identified poor and vulnerable populations; bottom-up planning to allow contextual pro-poor ACSM solutions in place

Decentralisation of DOT	Consider strengthening decentralisation; Innovations such as incentives for carrying drugs to health care workers/DOT providers where necessary to minimise drug shortages

Coverage: Population norms for designated microscopy centres (DMCs)	Consider optimising information relating to geography, physical distances, urban-rural differences, and access to transportation facilities. Allow flexibility to district programme managers where possible to ensure coverage approaches for TB control are pro-poor

Incentives for tribal areas available	Consider incentives for below-poverty line TB patients living in non-tribal areas in line with available incentives for poor in tribal areas. Also consider incentives for unemployed and poor living in urban slums

Honorarium to private provides to ensure DOT available	Ensure incentives through RNTCP schemes for sputum collection, transportation schemes etc. involving civil society partners and private sector

RNTCP schemes for NGOs and Private sector in urban slums	Consider implementing existing schemes optimally and modify incentives schemes where applicable based on experiences

**Table 5:** Equity mapping in TB control--possible indicators to measure equity: Recommendations from the deliberations of the Working groups

Specified groups	Possible indicators
Below Poverty Line	• Number of BPL chest symptomatics screened for tuberculosis/Total chest symptomatics suspects screened
	• Number of BPL screened for Extra-pulmonary TB/Total Extra-pulmonary TB suspects screened

Linkages with social welfare schemes	• Number of TB patients availing social welfare schemes/Total number of TB patients
	• Number of TB patients availing social welfare schemes/Total number of persons availing these schemes

PLWHA, Tobacco users, Diabetics, Drug abusers	• Number knowing their TB status/Total number of such patients

Primitive tribal groups (PTG)	• Number of PTG availing diagnostic services/Total population of PTG; Sputum positive cases/Total suspects screened

Chemoprophylaxis	• Number of eligible children receiving INH chemoprophylaxis/Total eligible children

Contact tracing	• Number of eligible contacts screened for TB/Total number of eligible contacts

Benatur et al. (2010) elucidate that extreme poverty along with malnutrition, overcrowding, and lack of access to health care are major drivers for tuberculosis and its spread, including its dangerous varieties namely multi-drug resistant and extremely drug resistant strains [15]. The role of disease control programmes such as RNTCP is central to addressing poverty today. An improvement in economy more recently witnessed in India has a potential to influence the incidence of tuberculosis in the country. Regression analysis suggests that with each doubling of GDP, there is an associated decrease of 38.5% in the incidence of TB [16]. While the poverty rates appear to have reduced, their absolute numbers increased due to the rising population in the country. Persisting inequity despite GDP growth and vulnerability with its newer variants emerge in the country [17]. While existing studies highlight the economic impact of the TB control efforts in India, it is crucial to note such assessments have not taken in to account the wider socio-economic disparities that prevail in the country.

The participants in the study discussed the barriers in accessing diagnostic and treatment services, especially among the poor and vulnerable populations within their jurisdictions. Several factors identified as impeding access to TB diagnostic services among the poor include lack of awareness of existing TB services, lower education levels among the marginalised groups, sub-optimal or ineffective ACSM implementation by the RNTCP, discrimination in relation to gender/age/religion, and apathy of health care providers towards the poor. Barriers to treatment services identified include suboptimal ACSM implementation by the RNTCP, lack of flexibility (timings/observer) in implementing DOTS including workplace DOTS, economic constraints faced by the poor, and lower incentive paid to DOT providers. Factors magnifying both diagnostic and treatment barriers included illiteracy, unemployment, lack of social security, migrants who legal status was unclear, gender and age related, location of health care services, lack of motivation among health care providers, added stigma of HIV and poor involvement of private providers.

Participants emphasised the need to strengthen the RNTCP's effort to engage and involve private providers, including informal providers, non-allopathic providers (AYUSH)^g ^to ensure providing access to TB related services targeting the poor. Line-listing of NGOs within identified poverty pockets followed by their sensitisation on available RNTCP schemes to promote their involvement will expand access to TB services within communities. Synergy with educational department by organising sensitization of school teachers and examining community-owned schemes such as Sarva Shiksha Abhiyan (SSA)^h ^were discussed. Collaboration with the national chambers of commerce such as the Confederation of Indian Industries (CII) to build synergy between corporate sector and RNTCP was considered essential to ensure work place DOTS. Public sector units (PSU) such as Indian Railways, Employees state insurance, Steel Authority of India (SAIL), Coal India (COAL), National Thermal Power Corporation (NTPC) have their own health facilities and establishing and strengthening linkages between PSU units and the RNTCP offered a possibility to widen reach and ensure optimising TB service provision to the poor.

RNTCP's revised scheme for NGOs and private providers introduced in 2008 has adopted innovative mechanisms to involve diverse stakeholders in TB control [18]. ACSM scheme, sputum collection scheme, transport scheme, treatment adherence scheme and urban slum schemes among others have been designed to improve access of TB services to the populations, especially the poor and needy. Special emphasis is provided within these schemes to engage institutions working in hard to reach areas or 'underserved' areas where there are challenges relating to accessing TB services. However, efficient uptake of these schemes will need concerted efforts on the part of the programme, as well as civil societies and private sector stakeholders concerned. There is limited awareness on the existence of these schemes among the stakeholders therefore calling for wider dissemination of schemes to promote stakeholder engagement.

Integrated Rural Development Programme or Swaranjayanti Gram Swarozgar Yojana (SGSY), Jawahar Rozgar Yojana, Rural Employment Generation Programme, Employment Assurance schemes, Sampoorna Grameen Rozgar Yojana (SGRY), Rural Housing- Indira Awaas Yojana, National Food for work programme, National Social Assistance Programme, Swarna Jayanti Shahri Rozgar Yojana and Old age pension scheme were some of the anti-poverty programmes being implemented by the government of India. Some of the above mentioned schemes may have merged with intent to promote concerted action at service delivery level. An inherent systemic challenge that may potentially hinder any synergistic efforts is the limited awareness regarding social welfare schemes among TB programme managers at state and district level, accompanied by perceived lack of coordination and linkages between the multi-department schemes and RNTCP. However what remains clear is that TB patients below the poverty line would benefit immensely if linkages between RNTCP and poverty alleviation schemes are established where they do not exist, and strengthen harmonization efforts where they already exist aimed at ultimately benefiting the poor. There was consensus that a detailed analysis of poverty alleviation programmes and examining their current and potential linkages with TB control is necessary if any attempt to promote synergistic efforts is envisioned.

## Conclusion

There are wide ranging interventions that were identified as pro-poor in the context of tuberculosis control. There is however very limited information on the effectiveness of these measures in reaching out to the poor. There is a need for evaluating and addressing wider issues related to poverty within the scope of the TB Control Programme.

Addressing health inequities necessitates multi-sectoral coordination, and that sustained TB control efforts involving pro-poor approaches with resulting decline in TB prevalence among the poor and advancing the welfare of the poor seems likely. This is possible only when intensified efforts sustained by the RNTCP, are augmented with coordinated and synergistic efforts of concerned departments across diverse sectors dealing with populations that are considered to be poor. However our current understanding of how tuberculosis control is advancing poverty alleviation efforts at the population level remains incomplete. The understanding, reflection and knowledge of the key stakeholders during this participatory workshop forms the basis for recommendations for action, further planning and research on pro-poor TB centric interventions in the country. An in-depth analysis and systems/policy/operations research exploring pro-poor initiatives as highlighted by stakeholders in this article, in particular examining service delivery synergies between existing poverty alleviation schemes and TB control programme aimed at benefiting poor and vulnerable populations is necessary.

## Limitations

The study findings are based on an agreed position and final consensus from participants participating in the study, but may not necessarily reflect the views of their respective organisations.

## Endnotes

^a ^Accredited social health activist--female community health activist trained to work as an interface between the community and the public health system.

^b ^Informal health provider--unqualified health service provider.

^c ^VHSC- created under national rural health mission, these committees have the responsibility for health at community level and receive untied grants for village level activities.

^d ^National rural health mission, launched by Ministry of Health and Family Welfare, Government of India envisages improving the availability of and access to quality health care by people, especially for those residing in rural areas, the poor, women and children.

^e ^RSBY launched by Ministry of Labour and Employment under Government of India provides health insurance coverage for Below Poverty Line (BPL) families, where beneficiaries are entitled to hospitalization coverage up to INR 30,000 for most of the diseases requiring hospitalization.

^f ^NREGA-Mahatma Gandhi National Rural Employment Guarantee Act, launched under the Ministry of Rural Development, Government of India aims at enhancing the livelihood security of people in rural areas by guaranteeing 100 days of wage-employment in a financial year to a rural household whose adult members volunteer to do unskilled manual work.

^g ^Ayurveda, Yoga & Naturopathy, Unani, Siddha and Homoeopathy (AYUSH).

^h ^Sarva Shiksha Abhiyan (SSA), Government of India's flagship programme for universal elementary education.

## Competing interests

The authors declare that they have no competing interests.

## Authors' contributions

Authors VK, NW were part of the writing team with contributions from AD, SS, SC, and LSC. VK drafted the manuscript, with manuscript review and edits by NW, SS, AD and SC. All authors read and approved the final manuscript.
